# Oral mucosal lesions and risk of all-cause and cardiovascular mortality in people treated with long-term haemodialysis: The ORAL-D multinational cohort study

**DOI:** 10.1371/journal.pone.0218684

**Published:** 2019-06-21

**Authors:** Marinella Ruospo, Suetonia C. Palmer, Giusi Graziano, Patrizia Natale, Valeria Saglimbene, Massimo Petruzzi, Michele De Benedittis, Jonathan C. Craig, David W. Johnson, Pauline Ford, Marcello Tonelli, Eduardo Celia, Ruben Gelfman, Miguel R. Leal, Marietta Török, Paul Stroumza, Luc Frantzen, Anna Bednarek-Skublewska, Jan Dulawa, Domingo del Castillo, Staffan Schön, Amparo G. Bernat, Jörgen Hegbrant, Charlotta Wollheim, Letizia Gargano, Giovanni F. M. Strippoli, on behalf of the ORAL-D Investigators

**Affiliations:** 1 Diaverum Medical Scientific Office, Lund, Sweden; 2 University of Otago, Christchurch, New Zealand; 3 University of Bari, Bari, Italy; 4 University of Sydney, Sydney, Australia; 5 Flinders University, Adelaide, Australia; 6 University of Queensland, Brisbane, Australia; 7 Translational Research Institute, Brisbane, Australia; 8 University of Calgary, Calgary, Canada; 9 Medical University of Lublin, Lublin, Poland; 10 Medical University of Silesia, Silesia, Poland; University of Wisconsin, UNITED STATES

## Abstract

**Background:**

Chronic kidney disease is a risk factor for oral diseases, which may be associated with premature death. We evaluated the risk of all-cause and cardiovascular mortality associated with oral mucosal lesions in adults with kidney failure treated with long-term haemodialysis.

**Methods:**

Oral mucosal lesions (herpes, ulceration, neoformation, white lesion, red lesion, oral candidiasis, geographical tongue, petechial lesions, and fissured tongue) were evaluated within the Oral Diseases in Haemodialysis (ORAL-D) study, a multinational cohort study of 4726 haemodialysis adults. We conducted cox regression analyses adjusted for demographic and clinical variables to evaluate the association with all-cause and cardiovascular mortality.

**Results:**

Overall, 4205 adults **(**mean age 61.6 ± 15.6 years) underwent oral mucosal examination with 40% affected by at least one lesion. The prevalence of oral lesions was (in order of frequency): oral herpes 0.5%, mucosal ulceration 1.7%, neoformation 2.0%, white lesion 3.5%, red lesion 4.0%, oral candidiasis 4.6%, geographical tongue 4.9%, petechial lesions 7.9%, and fissured tongue 10.7%. During median follow-up of 3.5 years, 2114 patients died (1013 due to cardiovascular disease). No association was observed between any individual oral lesion and all-cause or cardiovascular mortality when adjusted for comorbidities, except for oral candidiasis, which was associated with all-cause mortality (adjusted hazard ratio 1.37, 95% CI 1.00 to 1.86) and cardiovascular mortality (adjusted hazard ratio 1.64, 95% CI 1.09 to 2.46).

**Conclusion:**

Oral mucosal lesions are prevalent in haemodialysis patients. Oral candidiasis appears to be a risk factor for death due to cardiovascular diseases.

## Introduction

Patients with chronic kidney disease (CKD) may experience oral mucosal diseases more frequently than the general adult population [1].

A systematic review of observational data has shown a prevalence of 8.6% for mucosal ulceration, 2.5% for oral herpetic lesions, and 19.6% for oral candidiasis among people with kidney failure [1]. Recent studies have demonstrated that oral mucosal lesions are frequent in patients with CKD, including those receiving dialysis. Oral candidiasis is one of the most common forms of mucosal disease in this population [2][3]. However, knowledge about the prevalence and prognostic implications of oral mucosal lesions in the setting of dialysis is constrained by the existence of relatively few studies. Oral mucosal disease may reduce health-related quality of life and is increased among patients with long term conditions [4]. To date, the relationship of oral mucosal lesions with clinical outcomes, such as mortality, for people with CKD has not been investigated [5].

The Oral Diseases in Haemodialysis study (ORAL-D) was a prospective multinational cohort study involving 4205 participants treated with dialysis. ORAL-D was designed to evaluate the prevalence and severity of oral disease in adults treated with haemodialysis and the associations of oral disease and dental practices with all-cause and cardiovascular mortality [6]. Previous ORAL-D analyses have evaluated prevalence and mortality outcomes associated with dental disease and periodontitis [7–9]. The aim of this study was to evaluate the association of oral mucosal lesions with all-cause and cardiovascular mortality.

## Materials and methods

The reporting of this analysis follows the Strengthening the Reporting of Observational studies in Epidemiology (STROBE) guidelines (S1 Item) [10].

### Design and setting

We evaluated the point prevalence of oral mucosal lesions and their association with mortality in hemodialysis patients, as a substudy of ORAL-D, the methods of which have been reported previously [6–9]. In brief, the ORAL-D study involved a standardized oral and dental examination among 4726 unselected patients with kidney failure treated with long term hemodialysis in seven countries in Europe and South America. All participants provided written and informed consent before participation.

We received ethics approval for the ORAL Diseases in Hemodialysis (ORAL-D) study from the following responsible local Human Research Ethics Committees: Comitè de Protection des Personnes Sud-Medierranèe II reference number 2010-A01125-34 (France), Komisja Bioetyczna, Slaskiego Uniwersytetu Medycznego W Katowicach reference number KNW/0022/KB/204/10 (Poland), CE da Diaverum Portugal reference number 01/2010 (Portugal), Comite Etico de Investigacion Clinica (CEIC) de la Fundaction Puigvert reference number T2011/01 and Agencia Valenciana de Salud, Departament de Salut Valencia reference number 38/11 (Spain), and Szegedi Tudomanyegyetem, Szent-Gyorgyi albert klinikai kozpont, and Regionalis human orvosbiologiai kutatasetikai bizottsaga reference number 168/2009 (Hungary). Ethics approval was not required for this type of study in Italy or Argentina. The study was performed in accordance with the Declaration of Helsinki.

### Study population

Consecutive adults who were aged 18 years or older and treated with long-term hemodialysis for any duration were included from a convenience sample of clinics operated by a single dialysis provider in Europe (France, Hungary, Italy, Poland, Portugal, and Spain) and South America (Argentina). We enrolled participants between July 2010 and February 2012. Patients were not enrolled if they had cognitive impairment sufficient to preclude consent or if they preferred not to participate.

### Data sources

Demographic, clinical, laboratory and dialysis-related data were obtained from a centralized clinical database linked to particpants using a unique identification code. These data included age, sex, country, education level, marital and occupational status, alcohol intake, smoking history, physical activity, housing, family income, body mass index, comorbidities (including cardiovascular disease and diabetes), medication, serum parameters (including hemoglobin, phosphorus, parathyroid hormone, calcium, and albumin levels), and dialysis characteristics. Cause-specific mortality was ascertained and entered by treating clinicians on a monthly basis.

### Exposures

The exposure of interest was any oral mucosal lesion. All participants underwent a standardized oral examination before the start of a dialysis treatment by a trained dentist, according to a systematic approach recommended by the World Health Organization (WHO) [11]. Examining dentists participated in audio-teleconferences to discuss the study protocol and oral examination, as detailed previously [6]. The dentists recorded the presence of herpes, ulceration, neoformation, white lesion, red lesion, oral candidiasis, geographical tongue, petechial lesions and fissured tongue based on their clinical appearance. They were subsequently classified based on their infectious/traumatic nature. No tissue biopsies were taken. Patients with identified lesions requiring specific therapy were referred to a practitioner for diagnosis and treatment.

### Outcomes

The outcomes of interest were all-cause mortality and death due to cardiovascular causes. Cardiovascular death was defined as a sudden death or death due to acute myocardial infarction, pericarditis, atherosclerotic heart disease, cardiomyopathy, cardiac arrhythmia, cardiac arrest, valvular heart disease, pulmonary edema, congestive cardiac failure, cerebrovascular accident including intracranial haemorrhage, ischemic brain damage including anoxic encephalopathy, or mesenteric infarction or ischemia of the bowel. The cause of death was adjudicated by the usual attending physician who was not aware of the results of the oral examination.

### Statistical analysis

Baseline demographic, socioeconomic, clinical and dialysis characteristics were evaluated as frequencies (%), mean and standard deviation (SD), or medians with interquartile range (IQR). The prevalence of each mucosal lesion was calculated.

The point prevalence of oral mucosal lesions was assessed. Participant characteristics associated with the presence of mucosal lesions were evaluated using logistic regression. We then used a multivariable logistic model to include those covariates that were statistically significant in univariate models or were clinically relevant. Estimates were expressed as odds ratios (OR) and adjusted odds ratios (adjOR) together with their respective 95% confidence intervals (CI).

To estimate the association between mucosal lesions and all-cause and cardiovascular mortality, we used a Cox proportional hazard regression model adjusted for country, age, sex, education, smoking history, myocardial infarction, diabetes, hemoglobin, serum albumin, serum phosphorus, time on dialysis,body mass index, and any mucosal lesions (ulceration, white lesion, red lesion, neoformation, petechial lesions, geographical tongue, fissured tongue, oral candidiasis, and herpes). These covariates were included in the final model on the basis of clinical relevance or the statistical significance in univariate Cox analyses and were formally tested for collinearity. Backward elimination was then used for variable selection: we started by fitting a full model with all the variables listed above, then removing at each step the variable with the smallest contribution to the model and continuing until all remaining variables were statistically significant. The proportional hazards assumption was confirmed by fitting the interaction of selected covariates with time and graphically by plotting the log-negative-log of Kaplan-Meier estimator of the survival function.

Estimated mortality risks were expressed as hazard ratios (HR) and adjusted hazard ratios (adjHR) together with their respective 95% confidence intervals (CI). In addition, we used a random-effects Cox model fitted using shared frailty to account for clustering of mortality risk within countries. The Fine and Gray model was used to calculate the cumulative incidence function for cardiovascular mortality accounting for the competing risk of all-cause mortality. We repeated analyses restricted to participants with complete data for all variables. Statistical analyses were performed using SAS, version 9.4 (SAS Institute Inc).

## Results

**Fig 1:**
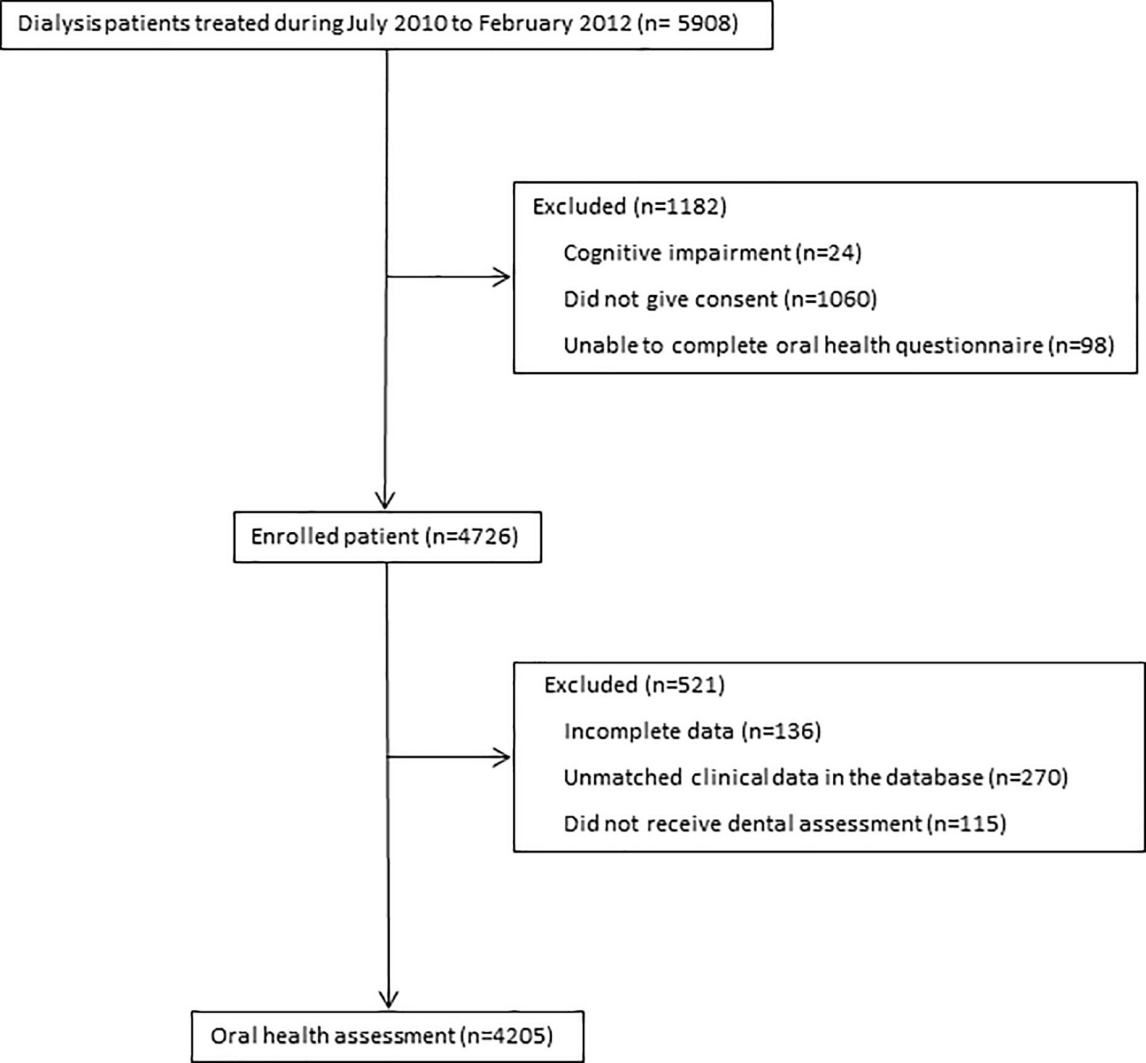
Flow chart of participation.

Overall, 4205 patients of the 4726 participants in ORAL-D (89%) underwent an examination for oral mucosal lesions between July 2010 and February 2012 and were included in the analysis (Fig 1 and Table 1). The mean age was 61.6 ± 15.6 years and 58% of participants were men. Overall, 1029 (33.4%) participants had never smoked.

**Table 1:** Characteristics of study participants.

**Demographics**	
Age, years	61.6 ± 15.6
Country	
Italy	593 (14.1)
France	48 (1.1)
Spain	189 (4.5)
Portugal	762 (18.1)
Argentina	1744 (41.5)
Hungary	550 (13.1)
Poland	319 (7.6)
Male	2426 (57.7)
European	2461 (58.5)
Ever smoker	1029 (33.4)
**Socioeconomic characteristics**	
Married	1660 (53.9)
Secondary education	1139 (37.6)
Employed	387 (12.6)
Family income > domestic average	313 (8.02)
BMI	25.8 ± 5.1
**Comorbidity conditions at baseline**	
Myocardial infarction	348 (12.6)
Stroke	287 (10.4)
Diabetes mellitus	933 (32.0)
**Laboratory variables**	
Serum albumin, g/L	38.3 ± 4.0
Serum phosphate, mg/dL	4.7 ± 1.5
Serum calcium, mg/dL	8.8 ± 0.8
Haemoglobin, g/dL	11.1 ± 1.3
**Dialysis variables**	
Time on dialysis (months)	60 (38–98)
Kt/V	1.7 ± 0.3
Urea reduction ratio	0.8 ± 0.1
Mean arterial pressure, mm Hg	90.0 ± 13.6
**Mucosal lesions frequency**	
Herpes	0.5 (0.3–0.8)
Ulceration	1.7 (1.3–2.1)
Neoformation	2.0 (1.6–2.5)
White lesion	3.5 (3.0–4.1)
Red lesion	4.0 (3.5–4.7)
Oral candidiasis	4.6 (4.0–5.2)
Geographical tongue	4.9 (4.3–5.6)
Petechial lesions	7.9 (7.1–8.7)
Fissured tongue	10.7 (9.8–11.7)

Results are expressed as number (%) for categorical variables and as mean ± SD or median (25^th^, 75^th^ centile) for continuous variables. Frequencies are expressed as proportion with 95% confidence interval.

### Prevalence

Forty percent of participants were affected by the presence of at least one oral lesion. In ascending order of frequency, 21 (0.5%) participants had oral herpes, 70 (1.7%) had mucosal ulceration, 85 had neoformation (2.0%), 147 (3.5%) white lesion, and 169 (4.0%) red lesion. Oral candidiasis was observed in 192 (4.6%) participants, geographical tongue in 207 (4.9%) participants, petechial lesions in 331 (7.9%), and fissured tongue in 450 (10.7%) participants.

In analyses adjusted for demographic and clinical characteristics, there was no statistically significant association between participant demographic and clinical variables and oral mucosal lesions (S1 and S2 Tables). The prevalence of oral mucosal lesions (especially geographical tongue, fissured tongue, and candidiasis) varied significantly by country. Oral mucosal lesions tended to be more common among patients treated in Italy, Poland and Spain (S1 Table).

### Mortality

**Fig 2:**
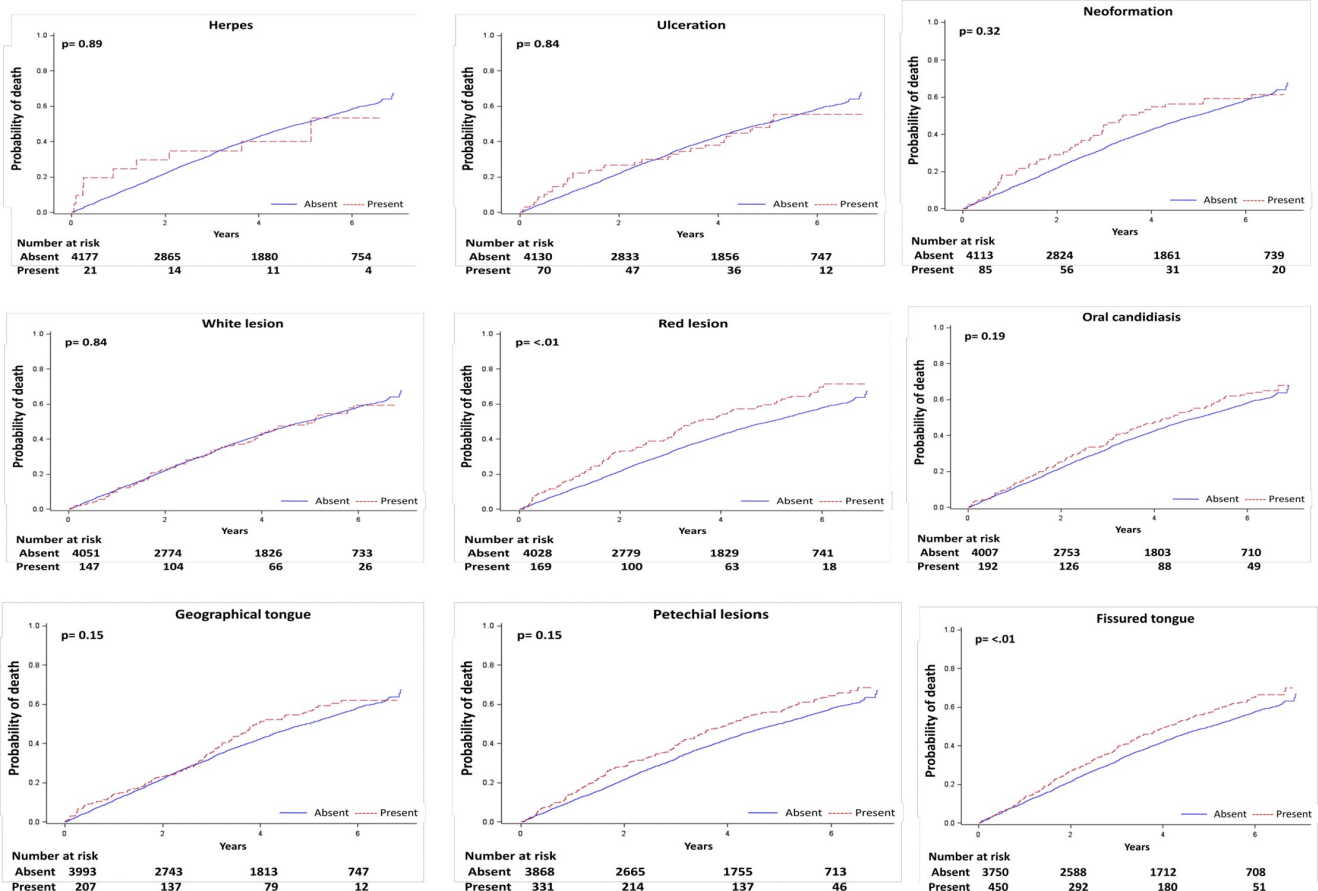
Unadjusted cumulative incidence of all-cause mortality for each mucosal lesion.

**Fig 3:**
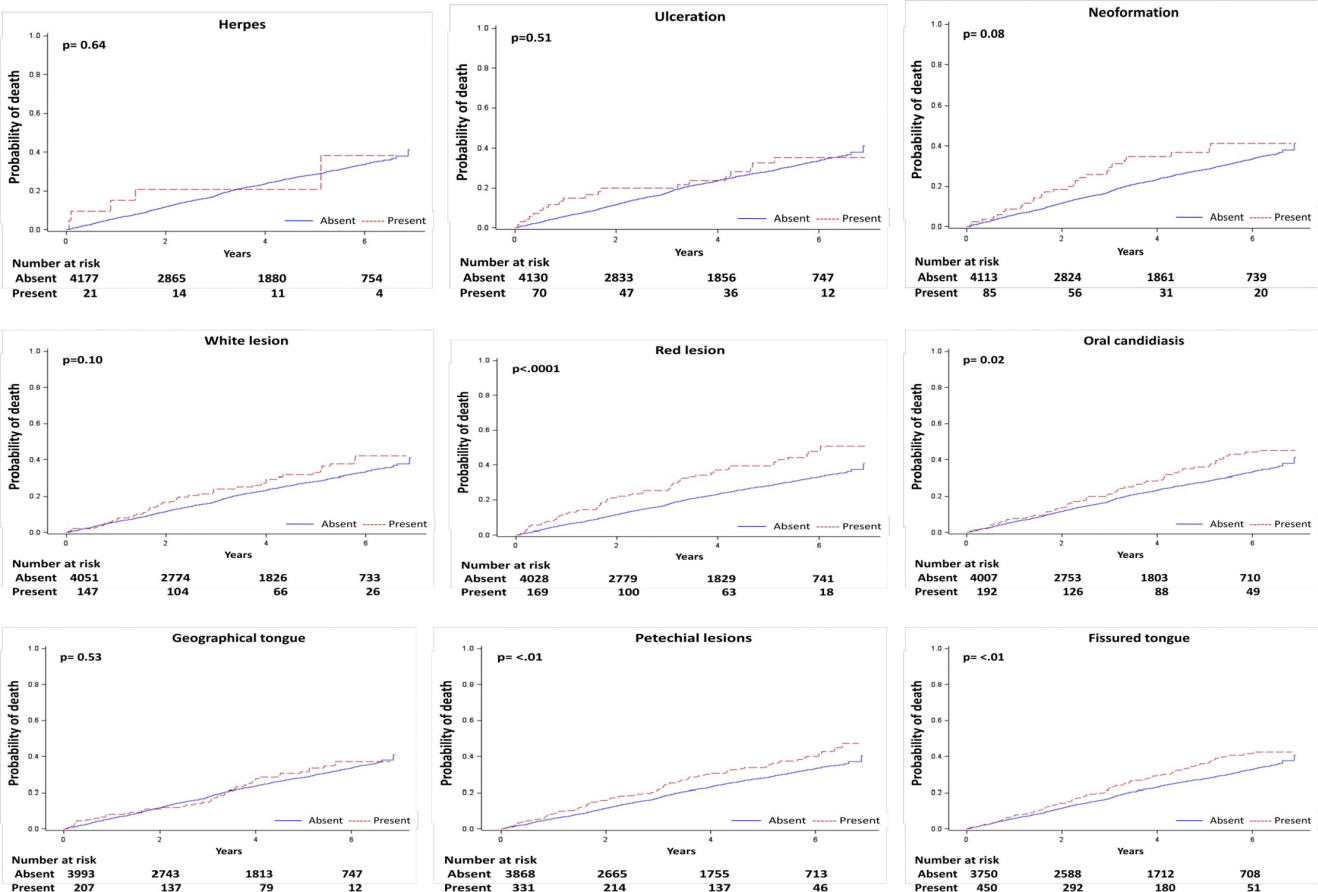
Unadjusted cumulative incidence of cardiovascular mortality for each mucosal lesion.

During a median follow-up of 3.5 (range, 1.6–5.8) years, 2114 (50%) deaths occurred of which 1013 (24%) were due to cardiovascular causes. As shown in Figs 2 and 3, red lesion, fissured tongue, and petechial lesions were associated with all-cause mortality in unadjusted analyses, while red lesion, fissured tongue, petechial lesions, and oral candidiasis were associated with cardiovascular death.

Following multivariable adjustment, there was no association between individual oral mucosal lesions and all-cause and cardiovascular death, except for oral candidiasis (Table 2), which was statistically significantly associated with all-cause (adjusted hazard ratio 1.37, 95% CI 1.00–1.86) and cardiovascular mortality (adjusted hazard ratio 1.64, 95% CI 1.09–2.46).

**Table 2:** All-cause and cardiovascular mortality associated with oral mucosal lesions.

	Death (N, %)		Unadjusted HR (95% CI)		Adjusted HR (95% CI) *	
Lesion (N patients)	All-cause	Cardiovascular	All-cause	Cardiovascular	**All-cause**	**Cardiovascular**
Herpes (21)	10 (48)	6 (29)	0.96 (0.52–1.79)	1.21 (0.54–2.70)	1.48 (0.54–4.03)	1.10 (0.26–4.58)
Ulceration (70)	35 (50)	20 (29)	0.97 (0.69–1.35)	1.16 (0.74–1.81)	1.09 (0.65–1.84)	1.17 (0.55–2.51)
Neoformation (85)	47 (55)	27 (32)	1.16 (0.87–1.55)	1.40 (0.96–2.05)	1.55 (0.94–2.55)	1.69 (0.80–3.59)
White lesion (147)	74 (50)	46 (31)	0.98 (0.78–1.23)	1.28 (0.96–1.73)	0.90 (0.60–1.35)	0.93 (0.52–1.63)
Red lesion (169)	100 (59)	58 (34)	1.39 (1.13–1.69)	1.69 (1.29–2.20)	0.98 (0.69–1.38)	1.07 (0.67–1.71)
Oral candidiasis (192)	111 (58)	62 (32)	1.14 (0.94–1.38)	1.34 (1.04–1.74)	1.37 (1.00–1.86)	1.64 (1.09–2.46)
Geographical tongue (207)	107 (52)	49 (24)	1.16 (0.95–1.40)	1.10 (0.82–1.46)	0.86 (0.62–1.18)	0.93 (0.57–1.54)
Petechial lesions (331)	190 (57)	98 (30)	1.24 (1.07–1.44)	1.34 (1.09–1.65)	1.12 (0.87–1.44)	1.01 (0.68–1.51)
Fissured tongue (450)	251 (56)	129 (29)	1.22 (1.07–1.39)	1.32 (1.10–1.59)	1.16 (0.93–1.45)	1.15 (0.84–1.59)

*The multivariable model was adjusted for country, age, sex, education, smoking history, myocardial infarction, diabetes, haemoglobin, serum albumin, serum phosphorus, time on dialysis, body mass index and oral mucosal lesions. HR: hazard ratio; CI: confidence interval.

### Sensitivity and subgroup analyses

Comparable results were observed when the cumulative incidence of cardiovascular death was calculated after modelling other causes of death as competing risks (S2 Table). When the analyses were conducted using shared frailty models to account for heterogeneity in survival between countries, the results were substantively similar (S3 Table).

## Discussion

In the ORAL-D, a multinational prospective cohort study in haemodialysis patients, oral mucosal lesions affected 40% of patients. Oral candidiasis was associated with increased all-cause and cardiovascular mortality after controlling for clinical and demographic factors. There was no evidence of increased all-cause or cardiovascular mortality associated with other oral mucosal lesions.

The finding of oral candidiasis in 4.6% of patients with kidney failure is similar to the finding of oral candidiasis in 7% of patients with Human Immunodeficiency Virus (HIV) infection, predominantly among patients with more advanced immunosuppression and higher viral load [12]. The similar prevalence of oral candida in HIV and kidney failure suggests a potential association with the lower immune function found in both conditions [13]. Notably, the point prevalence of oral ulceration, herpes, red and white lesions, and tongue changes were several-fold higher in the present cohort than observed in a general population characterized in the Third National Health and Nutrition Examination Survey from 1988–1994, suggesting mucosal changes may be more common with kidney failure [14]. This observation is consistent with our finding of a higher prevalence of dental conditions in end-stage kidney disease [7–9].

The observation that oral candidiasis is associated with mortality is consistent with prior studies showing an elevated risk for both all-cause mortality and cardiovascular mortality in patients with kidney failure who have other oral conditions, including tooth loss and dental caries. Oral candidiasis is an opportunistic infection of the mouth and is associated with steroid or antibiotic therapy, intercurrent comorbidity, diabetes, denture wearing and low salivary flow. Many of these features are present among people with kidney failure and are independently associated with poorer outcomes.

It is unclear in the present study whether oral candidiasis is causal in pathways for mortality or a confounding factor indicative of other comorbidities that represent the true aetiology of worse clinical outcomes, or an epiphenomenon occurring simultaneously with kidney failure but not necessarily caused by the condition. No association was found between any oral mucosal lesion and any socio-demographic factor. This finding is consistent with observations in an Australian National Survey of Adult Oral Health showing no relationship between oral mucosal lesions and social or clinical factors and may explain the lack of an association of non-candidiasis lesions with mortality in this dialysis population [15]. Alternative explanations for a lack of statistical association between oral mucosal lesions with mortality is the relatively low prevalence of many oral mucosal lesions, which may have led to imprecision in the estimated mortality risk. Alternatively, the absence of a statistical association may reflect a difference in the pathobiology of lesions and the predominant causes of mortality in the presence of kidney failure.

While these observations suggest there is limited association of oral mucosal lesions with mortality outcomes in dialysis patients, this finding does not preclude other negative consequences of oral mucosal lesions for patients with kidney failure. Notably, this study indicates that approximately 5–10% of dialysis patients experience clinical mucosal lesions at any given time. Based on existing evidence, it is probable that oral lesions have a negative impact on oral-specific and general quality of life for dialysis patients [16]. Whether oral lesions lead to important functional limitations such as discomfort while eating, physical pain, taste, and speech articulation in dialysis patients warrants investigation.

Our finding of an association between oral candidiasis and increased mortality in hemodialysis patients suggests that prevention or treatment of oral mucosal lesions might improve clinical outcomes in this population. However, this hypothesis needs to be tested in a sufficiently powered randomized controlled trial along with demonstrated feasibility of screening and treatment for oral candidiasis in hemodialysis patients.

The strengths of this study include a large multinational sample size, inclusion of representative patients in typical dialysis clinics, and inclusion of routine data collection for potential confounding factors such as financial status and education. Our study has potential limitations. First, due to the observational study design, this analysis cannot evaluate any causal impact of oral mucosal lesions on mortality risk. Although there was adjustment for an extensive number of potential confounders, the risk of residual confounding is still possible. Second, we evaluated all-cause mortality and cardiovascular mortality as key outcomes. While there was no evidence of an association for these outcomes, we did not evaluate linkages between oral mucosal lesions and other patient-important factors, including pain, health-related quality of life, nutrition, and oral function. Third, despite a higher prevalence of oral mucosal lesions than has been reported in the general population, the relatively few participants who had oral mucosal lesions may have reduced the power in the analyses to detect significant associations between mucosal lesions and mortality. Fourth, oral candidiasis and herpetic lesions were assessed clinically and did not include microbiological testing or evaluation of type (e.g. pseudomembranous or erythematous candidiasis), leading to the possibility of measurement error. Fifth, we observed different prevalence rates of oral mucosal lesions across the different countries contributing data. While the dental examination was conducted using a standardized approach, differences in prevalence may have represented variation in diagnostic practices in each country.

In conclusion, the prevalence of oral mucosal lesions may be higher in dialysis patients than in the general population, although such lesions appear unrelated to measured sociodemographic and clinical characteristics. Oral candidiasis was present in 5% of this dialysis population and was associated with all-cause and cardiovascular mortality.

## Supporting information

S1 Item STROBE statement—Checklist of items that should be included in reports of observational studies. (PDF)

S1 Table Association of clinical and demographic variables with mucosal lesions. (PDF)

S2 Table Association of mucosal lesions with cardiovascular mortality accounting for all-cause mortality as competing risk. (PDF)

S3 Table Association of mucosal lesions with all-cause and cardiovascular disease using a Cox proportional hazards model fitted with shared frailty to account for within-country clustering of mortality risks. (PDF)
