# Supplementary material for: Oral mucosal lesions and risk of all-cause and cardiovascular mortality in people treated with long-term haemodialysis: The ORAL-D multinational cohort study
Source: PLoS One. 2019 Jun 21;14(6):e0218684. doi: 10.1371/journal.pone.0218684 (PMC6588239; doi:10.1371/journal.pone.0218684)
Supplement: S1 Item — (PDF) [file pone.0218684.s001.pdf]

S1 Item. STROBE Statement—checklist of items that should be included in reports of observational studies

	Item No	Recommendation	Page(s)
**Title and abstract**	1	(*a*) Indicate the study's design with a commonly used term in the title or the abstract	Title page
		(*b*) Provide in the abstract an informative and balanced summary of what was done and what was found	Page 3
**Introduction**			
Background/rationale	2	Explain the scientific background and rationale for the investigation being reported	Page 5
Objectives	3	State specific objectives, including any prespecified hypotheses	Page 5
**Methods**			
Study design	4	Present key elements of study design early in the paper	Pages 6
Setting	5	Describe the setting, locations, and relevant dates, including periods of recruitment, exposure, follow-up, and data collection	Page 6-7
Participants	6	(*a*) *Cohort study*—Give the eligibility criteria, and the sources and methods of selection of participants. Describe methods of follow-up	Page 6-7
		(*b*) *Cohort study*—For matched studies, give matching criteria and number of exposed and unexposed	NA
Variables	7	Clearly define all outcomes, exposures, predictors, potential confounders, and effect modifiers. Give diagnostic criteria, if applicable	Page 7
Data sources/ measurement	8*	For each variable of interest, give sources of data and details of methods of assessment (measurement). Describe comparability of assessment methods if there is more than one group	Page 6-7
Bias	9	Describe any efforts to address potential sources of bias	Pages 6-8
Study size	10	Explain how the study size was arrived at	See study design publication, ref. 5
Quantitative variables	11	Explain how quantitative variables were handled in the analyses. If applicable, describe which groupings were chosen and why	NA
Statistical methods	12	(*a*) Describe all statistical methods, including those used to control for confounding	Pages 8-9
		(*b*) Describe any methods used to examine subgroups and interactions	Pages 8-9
		(*c*) Explain how missing data were addressed	Pages 8-9
		(*d*) *Cohort study*—If applicable, explain how loss to follow-up was addressed	
		(*e*) Describe any sensitivity analyses	Page 8-9

Continued on next page

Page(s)

**Results**			
Participants	13*	(a) Report numbers of individuals at each stage of study—eg numbers potentially eligible, examined for eligibility, confirmed eligible, included in the study, completing follow-up, and analysed	Pages 9-10
		(b) Give reasons for non-participation at each stage	Figure 1
		(c) Consider use of a flow diagram	Figure 1
Descriptive data	14*	(a) Give characteristics of study participants (eg demographic, clinical, social) and information on exposures and potential confounders	Table 1
		(b) Indicate number of participants with missing data for each variable of interest	Table 1
		(c) *Cohort study*—Summarise follow-up time (e.g., average and total amount)	Page 10
Outcome data	15*	*Cohort study*—Report numbers of outcome events or summary measures over time	Page 10
Main results	16	(*a*) Give unadjusted estimates and, if applicable, confounder-adjusted estimates and their precision (e.g., 95% confidence interval). Make clear which confounders were adjusted for and why they were included	Page 8 and supplementary material
		(*b*) Report category boundaries when continuous variables were categorized	NA
		(*c*) If relevant, consider translating estimates of relative risk into absolute risk for a meaningful time period	Page 10 and supplementary material
Other analyses	17	Report other analyses done—eg analyses of subgroups and interactions, and sensitivity analyses	Page 10 and supplementary material
**Discussion**			
Key results	18	Summarise key results with reference to study objectives	Page 11
Limitations	19	Discuss limitations of the study, taking into account sources of potential bias or imprecision. Discuss both direction and magnitude of any potential bias	Page 11-13
Interpretation	20	Give a cautious overall interpretation of results considering objectives, limitations, multiplicity of analyses, results from similar studies, and other relevant evidence	Page 11-13
Generalisability	21	Discuss the generalisability (external validity) of the study results	Page 11-13
**Other information**			
Funding	22	Give the source of funding and the role of the funders for the present study and, if applicable, for the original study on which the present article is based	Page 14
