# Supplementary material for: Oral mucosal lesions and risk of all-cause and cardiovascular mortality in people treated with long-term haemodialysis: The ORAL-D multinational cohort study
Source: PLoS One. 2019 Jun 21;14(6):e0218684. doi: 10.1371/journal.pone.0218684 (PMC6588239; doi:10.1371/journal.pone.0218684)
Supplement: S1 Table — (PDF) [file pone.0218684.s002.pdf]

S1 Table. Association of clinical and demographic variables with mucosal lesions

	Herpes	Ulceration	Neoformation	White lesion	Red lesion	Oral candidiasis	Geographical tongue	Petechial lesions	Fissured tongue
	Adjusted odds ratio (95% confidence interval)								
**Age**	0.98(0.94-1.02)	1.00(0.97-1.03)	1.02(0.99-1.05)	1.01(0.98-1.03)	1.03(1.01-1.06)	1.00(0.98-1.02)	1.01(1.00-1.03)	1.02(1.01-1.04)	1.01(1.00-1.03)
**Sex**									
**Female**	reference	reference	reference	reference	reference	reference	reference	reference	reference
**Male**	1.98(0.59-6.62)	1.2(0.54-2.69)	1.15(0.57-2.3)	1.68(0.85-3.34)	0.73(0.41-1.3)	1.89(1.04-3.43)	0.66(0.39-1.11)	1.26(0.81-1.98)	1.32(0.9-1.94)
**Country**									
**Portugal**	reference	reference	reference	reference	reference	reference	reference	reference	reference
**Argentina**	0.07(0.00-1.32)	0.55(0.12-2.44)	0.20(0.04-1.09)	1.23(0.39-3.86)	31.7(2.05-491)	0.43(0.07-2.85)	13.1(4.18-41.0)	0.47(0.23-0.93)	3.54(1.40-8.90)
**France**	-	7.36(0.23-238)	5.70(0.20-162)	5.17(0.17-160)	-	7.30(0.22-238)	17.46(0.56-541)	1.02(0.04-29.0)	19.01(1.84-196)
**Hungary**	1.00(0.07-15.2)	3.63(1.29-10.2)	4.85(1.92-12.2)	6.46(2.55-16.3)	-	0.96(0.15-6.21)	1.29(0.26-6.40)	0.74(0.39-1.41)	33.5(14.5-77.4)
**Italy**	1.68(0.24-11.8)	0.83(0.23-2.98)	2.05(0.80-5.21)	1.46(0.50-4.26)	5.87(0.32-108)	28.1(9.67-81.8)	1.60(0.41-6.34)	0.3(0.14-0.61)	7.08(2.99-16.7)
**Poland**	2.33(0.10-53.2)	2.68(0.45-16.1)	5.86(1.55-22.1)	6.86(2.05-22.9)	-	53.8(15.8-182)	5.64(1.09-29.0)	5.56(2.72-11.4)	13.8(4.55-41.7)
**Spain**	22.6(4.72-108)	3.76(1.30-10.8)	1.01(0.25-4.11)	6.25(2.33-16.7)	-	5.81(1.59-21.2)	67.6(22.9-199)	4.60(2.77-7.64)	43.4(18.7-100)
**Education**									
**0-8 years**	reference	reference	reference	reference	reference	reference	reference	reference	reference
**>8 years**	0.55(0.15-2.02)	0.53(0.23-1.21)	0.62(0.31-1.28)	1.46(0.81-2.63)	1.01(0.56-1.82)	0.88(0.52-1.50)	1.22(0.73-2.06)	1.00(0.65-1.52)	1.21(0.83-1.76)
**Smoking status**									
**Never smoked**	reference	reference	reference	reference	reference	reference	reference	reference	reference
**Ever smoker**	0.57(0.15-2.09)	1.88(0.89-3.97)	0.87(0.42-1.83)	1.71(0.95-3.06)	0.88(0.48-1.63)	1.26(0.74-2.14)	1.34(0.82-2.19)	1.06(0.70-1.61)	1.20(0.82-1.75)
**Myocardial infarction**									
**No**	reference	reference	reference	reference	reference	reference	reference	reference	reference
**Yes**	0.47(0.04-5.07)	1.51(0.64-3.59)	0.83(0.34-2.04)	1.04(0.51-2.13)	1.26(0.62-2.60)	1.04(0.55-1.96)	0.83(0.39-1.77)	1.81(1.14-2.87)	1.15(0.72-1.84)
**Diabetes**									
**No**	reference	reference	reference	reference	reference	reference	reference	reference	reference
**Yes**	0.11(0.01-1.05)	0.71(0.33-1.51)	1.09(0.56-2.11)	2.16(1.23-3.79)	0.48(0.26-0.88)	1.58(0.95-2.62)	0.78(0.48-1.28)	0.92(0.62-1.38)	0.98(0.68-1.41)
**Serum albumin, g/L**	0.81(0.69-0.93)	0.99(0.90-1.09)	0.95(0.87-1.03)	0.99(0.92-1.08)	0.96(0.89-1.03)	0.94(0.88-1.01)	1.00(0.93-1.07)	0.96(0.91-1.01)	0.94(0.90-0.99)
**Kt/V**	3.42(0.54-21.7)	0.71(0.17-2.92)	1.67(0.52-5.38)	0.68(0.21-2.16)	0.95(0.33-2.71)	1.77(0.74-4.20)	0.54(0.23-1.28)	0.84(0.40-1.77)	2.41(1.29-4.53)
**Serum phosphorus, mg/dL**	1.57(1.05-2.35)	0.98(0.75-1.29)	1.06(0.83-1.36)	1.00(0.81-1.24)	1.01(0.82-1.23)	1.03(0.85-1.26)	0.93(0.79-1.09)	0.88(0.76-1.03)	1.05(0.92-1.20)

The multivariable model was adjusted for country, age, sex, education, smoking history, prior myocardial infarction, diabetes, serum albumin, serum phosphorus and Kt/V
