# Supplementary material for: Oral mucosal lesions and risk of all-cause and cardiovascular mortality in people treated with long-term haemodialysis: The ORAL-D multinational cohort study
Source: PLoS One. 2019 Jun 21;14(6):e0218684. doi: 10.1371/journal.pone.0218684 (PMC6588239; doi:10.1371/journal.pone.0218684)
Supplement: S3 Table — (PDF) [file pone.0218684.s004.pdf]

**S3 Table. Association of mucosal lesions with all-cause and cardiovascular disease using a Cox proportional hazards model fitted with shared frailty to account for within-country clustering of mortality risks**

| | All-cause mortality | Cardiovascular mortality |
|---|---|---|
| | Adjusted HR (95% confidence interval) | |
| Herpes | 1.47 (0.54-4.02) | 1.11 (0.27-4.63) |
| Ulceration | 1.10 (0.65-1.87) | 1.13 (0.52-2.47) |
| Neoformation | 1.55 (0.94-2.56) | 1.68 (0.79-3.59) |
| White lesion | 0.91 (0.60-1.36) | 0.93 (0.53-1.65) |
| Red lesion | 0.98 (0.70-1.39) | 1.09 (0.68-1.77) |
| Candidiasis | 1.29 (0.94-1.75) | 1.57 (1.04-2.36) |
| Geographical tongue | 0.85 (0.61-1.17) | 0.96 (0.58-1.58) |
| Petechial lesions | 1.10 (0.85-1.41) | 0.99 (0.66-1.47) |
| Fissured tongue | 1.18 (0.94-1.48) | 1.17 (0.84-1.61) |

The multivariable model was adjusted for country, age, sex, education, smoking history, prior myocardial infarction, diabetes, hemoglobin, serum albumin, serum phosphorus, time on dialysis, body mass index and any mucosal lesions
